# Clinical significance of T-bet, GATA-3, and Bcl-6 transcription factor expression in bladder carcinoma

**DOI:** 10.1186/s12967-016-0891-z

**Published:** 2016-05-30

**Authors:** Islem Ben Bahria-Sediki, Nadhir Yousfi, Catherine Paul, Mohamed Chebil, Mohamed Cherif, Rachida Zermani, Amel Ben Ammar El Gaaied, Ali Bettaieb

**Affiliations:** Laboratoire d’Immunologie et Immunothérapie des Cancers (LIIC), EPHE, PSL Research University, 75014 Paris, France; Université Bourgogne Franche-Comté, EA7269, 21000 Dijon, France; Laboratoire de Génétique, Immunologie et Pathologies Humaines, Faculté de Sciences Tunis, Université de Tunis El Manar, Tunis, Tunisia; Hôpital Charles Nicolle de Tunis, Tunis, Tunisia; UFR des Sciences de Santé, 7 boulevard Jeanne d’Arc, BP 87900, 21079 Dijon, France

**Keywords:** Bladder carcinomas, T-bet, GATA-3, Bcl-6, Prognosis

## Abstract

**Background:**

The aim of this study was to investigate the clinical significance of three immune cell-related transcription factors, T-bet, GATA-3 and Bcl-6 in bladder cancer in Tunisian patients.

**Methods:**

Expression of T-bet, GATA-3 and Bcl-6 genes was assessed using RT-qPCR in 65 bladder cancers from patients: 32 being diagnosed as low- and medium-grade, 31 as high-grade, 25 as muscle invasive stage and 39 as non-muscle invasive stage. Gene expression was statistically correlated according to the grade, the stage, tobacco consumption, the BCG response and disease severity.

**Results:**

T-bet levels in patients with high-grade bladder cancer were significantly elevated compared to patients with low- or medium-grade bladder cancer (p = 0.005). In invasive carcinoma (T2–T4), the T-bet levels were significantly higher than in superficial non-invasive bladder tumors (Tis, Ta, and T1) (p = 0.02). However, T-bet is predictive of the response to BCG. Its expression is high in good responders to BCG (p = 0.02). In contrast, the expression of GATA-3 and Bcl-6 in non-invasive carcinoma (p = 0.008 and p = 0.0003) and in patients with low- and medium-grade cancers (p = 0.001 and p < 0.0001) is significantly higher than in invasive bladder tumors and in patients with high-grade bladder carcinoma, respectively. In addition, heavy smokers, whose tumors express low levels of GATA-3 and Bcl-6, are poor responders to BCG (p = 0.01 and p = 0.03). Finally, better patient survival correlated with GATA-3 (p = 0.04) and Bcl-6 (p = 0.04) but not T-bet expression.

**Conclusions:**

Our results suggest that T-bet expression in bladder tumors could be a positive prognostic indicator of BCG therapy, even if high levels are found in high-grade and stage of the disease. However, GATA-3 and Bcl-6 expression could be considered as predictive factors for good patient survival.

## Background

Global analysis of cancers revealed the importance of the adaptive immune cell response against tumors for cancer patient survival. However, the clinical outcome depends on the nature, functional orientation, density and location of immune cells within the tumor microenvironment [1]. For instance, ample clinical evidence shows that longer survival of cancer patients is associated with increased expression of genes characteristic of type 1 effector T cells, in particular the T-box master transcription factor regulator (T-bet) [2, 3]. Although T-bet is the master regulator of Th1 cell differentiation, it is expressed in multiple cells of the innate and adaptive immune system, including innate lymphoid cells (ILCs) [4, 5]. CD8^+^-infiltrating lymphocytes are also predictive of survival in muscle-invasive urothelial carcinoma [6] and are associated with a favorable prognosis in ovarian cancer [7]. This contrasts with the accumulation of tumor-infiltrating regulatory T cells (Tregs), where it is attested that the master regulator of Treg differentiation Foxp-3 is expressed. This creates an immunosuppressive microenvironment leading to tumor progression [8]. This is due to the ability of Treg cells to dampen the activity of CD4^+^ and CD8^+^ T cells, as well as natural killer (NK) cells, mainly by the release of transforming growth factor β (TGFβ1) and IL-10 [9]. Other adaptive cells are differently associated to cancer patient outcome. The Th2 response, evidenced inter alia by the expression of GATA-3, was associated with tumor immune evasion in a mouse study [10], but was not associated with a clinical outcome in human studies [11, 12] or in patient’s prognosis [13]. Patients with a low Th17 response had a better disease-free survival [13], whereas Th17 cells increased with advanced gastric cancer [14]. The opposite effect was observed for ovarian cancer [15]. Concerning follicular helper T cells (Tfh), an additional effector subset of T helper lymphocytes whose development is controlled by the B-cell lymphoma 6 transcription factor (Bcl-6), the presence of Tfh was high in various human cancers including malignancies in the lymphoid system [16]. Where the Tfh presence is significant, it has been associated with a better patient cancer outcome (breast cancer) [17], whereas a decreased proportion of Tfh was associated with increased hepatocellular carcinoma disease progression [18].

In the field of bladder cancer, few studies have addressed the role of immune cells in anti-tumor or pro-tumor activity and the correlation between those infiltrates and the clinical outcome [6, 19, 20].

Bladder cancer is the ninth most common malignancy worldwide, with its highest incidence rates in Western countries [21]. In Tunisia, bladder cancer is the second cause of cancer in the Tunisian male. The most effective therapy against superficial bladder cancer is intra-vesicle infiltration with Bacillus Calmette Guérin (BCG) [22]. However, some recurrence and progression occur after this therapy, which renders the response to BCG unpredictable. Hence, it is necessary to pinpoint reliable predictive biomarkers that could identify groups at elevated risk of treatment failure for a specific therapy.

The aim of this study is to assess the prognostic value of three genes in tumors from bladder cancer patients in Tunisia: T-bet, GATA-3 and Bcl-6. We found that upon disease progression, patients with high expression levels of T-bet were related to invasive and high grade of the disease, but were good responders to BCG. In contrast, reductions in Bcl-6 and GATA-3 expression correlated with invasive and high-grade of bladder cancer and were associated with a decrease in disease-free survival. Our results suggest that Th1−, Th2− and Tfh− associated gene expression could represent potential prognostic markers in patients with bladder cancer.

## Methods

### Patients and samples

A total of 65 patients with bladder carcinoma, for whom transurethral resections were realized at the Urology Department of Charles Nicolle Hospital (Tunis, Tunisia) between 1999 and 2012. Informed consent was obtained from all patients before their enrolment in the study. This study is in agreement with the Declaration of Helsinki and was approved by the Ethical Committee of the Tunis Pasteur Institute. All patients were from the North of Tunisia: 93.84 % of them were men and 6.15 % were women, and the mean age at diagnosis was 65.63 ± 13.13 years. Histopathological grading and staging were performed according to the WHO and TNM classification, respectively (Table 1).

**Table 1:** Characteristics of TCC tumor samples and patient information

Characteristics	Number of patients (n = 65)
Mean age (years)	
Median	65.63
Range	42–107
Gender	
Male	(93.85 %)
Female	(6.15 %)
Tumor stage	
Superficial (pta_:_ ptl_,_ TIS)	39 (60.00 %)
Invasive (pt>2)	25 (38.46 %)
Unknown	1 (1.54 %)
Tumor grade	
Low-grade (GI)	24 (37.50 %)
Medium-grade (GII)	8 (12.50 %)
High-grade (GIII)	31 (48.44 %)
Unknown	1 (1.56 %)
Bladder cancer recurrence	
Recurrence	28 (49.12 %)
No recurrence	29 (50.88 %)
Bladder cancer progression	
Progression	11 (39.29 %)
No progression	17 (60.71 %)

*TCC* transitional cell carcinoma

### RNA extraction from tumors

Bladder tumor samples ranging from stage and grade according to the TNM classification were obtained after surgical removal and 65 tissues samples were stored at −80 °C until the extraction of RNA. Total RNA from tumor tissue was isolated with TRIzol reagent (Invitrogen) according to the manufacturer’s protocol. After determination of the quality and concentration of the RNA by measuring optical density values at 260 and 280 nm with a nano-Drop ND-1000 Spectrophotometer (NanoDrop Technologies), the RNA was stored at −80 °C until use.

### RT-qPCR analysis

The mRNA levels of three genes, namely T-bet, GATA-3, and Bcl-6, were determined by quantitative real-time reverse-transcriptase PCR (RT-qPCR). In a first step, complementary DNA (cDNA) was reverse transcribed from one microgram (1 μg) of total RNA, using a random oligonucleotide primer and M-MLV reverse transcriptase (Promega, USA), according to the manufacturer’s instructions. The cDNA was stored at −20 °C until use. For real-time PCR the SYBR Green PCR master mix (Applied Biosystems by life technologies) was used. PCR reactions were carried out on a 7500 Fast Real-Time PCR System (Applied Biosystems) using the following conditions: an initial incubation at 50 °C for 2 min then 95 °C for 10 min, followed by 45 cycles of 95 °C for 15 s and 60 °C for 1 min. L32 mRNA was measured as the endogenous control (reference gene). All reactions were performed in triplicate. Primers for the amplification of the T-bet, GATA-3, Bcl-6, and L32 genes are described in Table 2, and were from Eurogentec.

**Table 2:** Sequences of primers for RT-PCR

Target gene	Oligonucleotide sequence (5ʹ→ 3ʹ)
L32	F- CTG CAG TCT CCT TGC ACA CTT
	R-TCT CCT GAA TGT GGT CAC CTG A
T-bet	F-CCC CTT GGT GTG GAC TGA GA
	R-ACG CGC CTC CTC TTA GAG TC
GATA-3	F-GTC CTC CCT GAG CCA CAT CT
	R-GTG GTC CAA AGG ACA GGC TG
Bcl-6	F-ACC TGC GAA TCC ACA CAG GA
	R-AGT CGC AGC TGG CTT TTG TG

*F* forward primer, *R* reverse primer

Relative mRNA levels for each sample were quantified using the Ct approach (fluorescence threshold), normalized to L32 mRNA as the standard. Expression of L32 was used as an endogenous reference and expression of T-bet, GATA-3 and Bcl-6 target genes in each sample were normalized to the mean of all Ct (Ct Calib). Thus, the relative gene expression level was calculated as the normalized relative quantity:

$${\text{NRQ}} \, = \,\frac{{ 2^{{\left( {\Delta {\text{CT target gene}}} \right)}} }}{{ 2^{{(\Delta {\text{CT L32}})}} }}$$

where ∆Ct target gene = Ct (calib-target gene)−Ct target gene and ∆Ct L32 = Ct (calib-L32)−Ct L32

### Statistical analysis

For basic statistical calculations, all gene expression levels were treated as continuous variables. Differences in gene expression between different clinical statuses were analyzed by the Student test using Graph pad prism 6. The nonparametric Mann–Whitney U test was used for statistical evaluation of the differences between two independent groups. Survival time was analyzed using Kaplan–Meier curves. Overall survival (OS) was also compared using the log-rank and the Gehan–Wilcoxon tests. Differences were considered statistically significant when the p-value was <0.05.

## Results

### Outcome of patients upon surgery

**Fig. 1:**
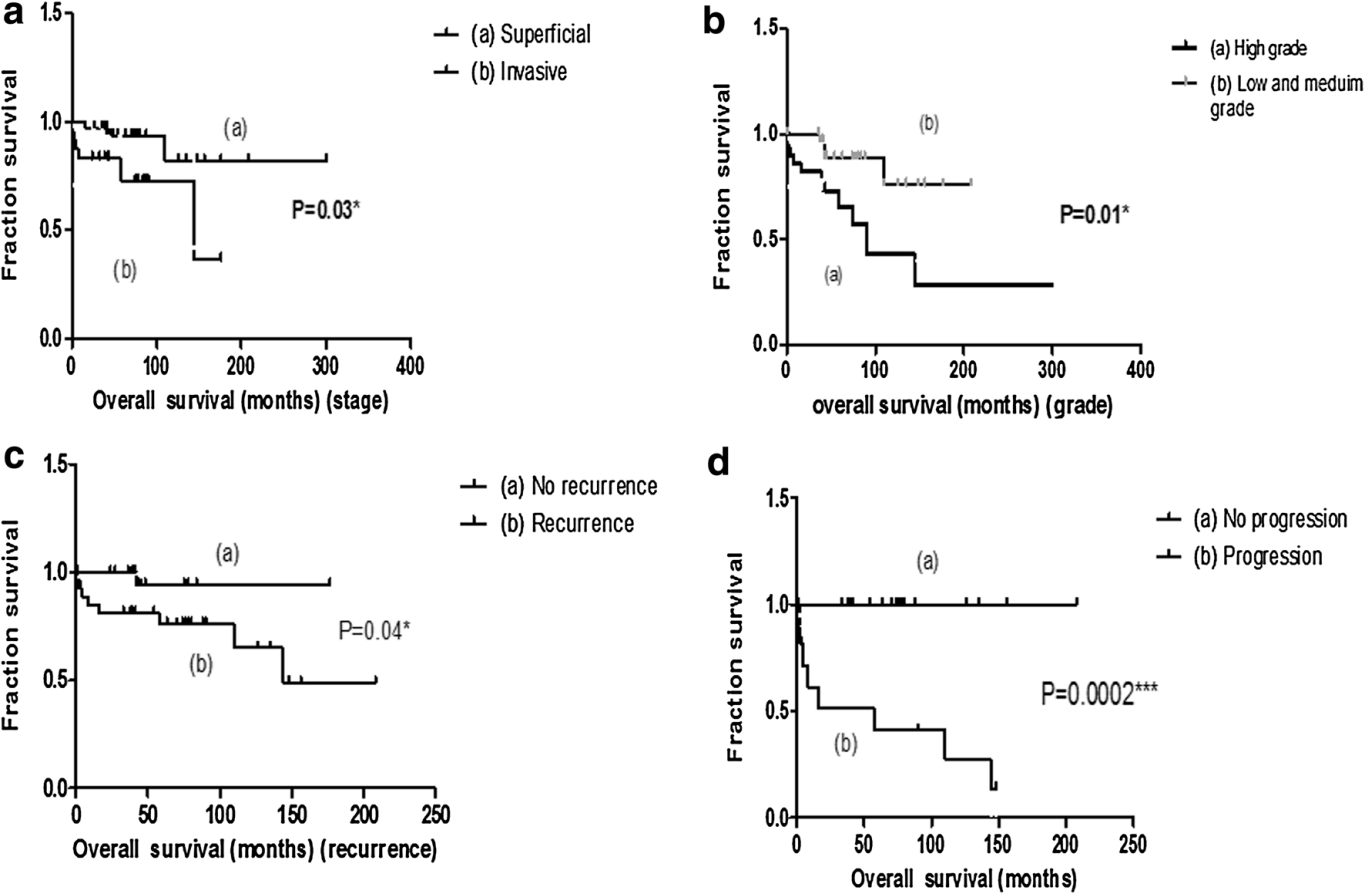
Kaplan–Meir plots for overall survival (*OS*) according clinic-pathological feature in patients with primary bladder cancer. Stratified survival curves for 65 patients were evaluated. **a** Patients with superficial and invasive stage *p = *0.03.* **b** Patients with low and medium- and high- grade tumors *p = *0.01.* **c** Patients with and without recurrence history *p = *0.04*. **d** Patients with and without tumor progression ***p = *0.0002*

The OS of our cohort of patients with bladder carcinoma was determined according to clinic-pathological features. As expected, little or no mortality was observed in patients without recurrence and disease progression, compared to those with recurrence and progression (Fig. 1a, b). Similarly, patients with superficial and low- or medium-grade cancers had prolonged disease-free survival compared to those with invasive or high-grade disease (Fig. 1c, d).

### T-bet expression in bladder tumor

**Fig. 2:**
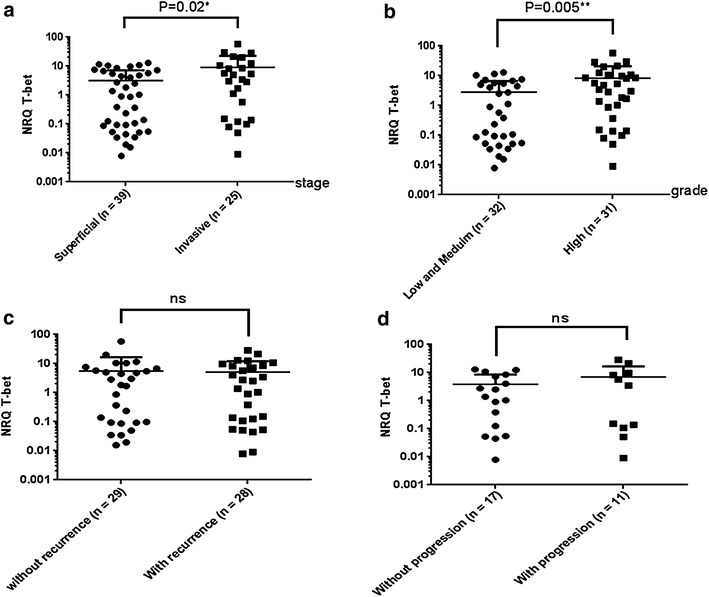
Relative expression of T-bet in primary tumor biopsies. Histogram showing the expression levels of T-bet according to clinic-pathological features: **a** Statistical analyses of T-bet expression between superficial and invasive histological stage of bladder cancer *p = *0.02*. **b** Statistical analyses of T-bet expression between low and high histological grade of bladder cancer **p = *0.005*. **c** No correlation of T-bet expression according to recurrence. **d** No correlation of T-bet expression according to tumor progression. L32 was used as the endogenous reference, and the expression of T-bet target gene in each sample was normalized (as described in “Methods” section). All PCR samples were assessed in triplicate

Expression of the Th1 transcription factor T-bet in bladder carcinoma was determined by real-time PCR as a function of the TNM histological stage of the disease. In invasive carcinoma (T2–T4), the level of T-bet was significantly higher (p = 0.02) than in superficial non-invasive bladder tumors (Tis, Ta, and T1) (Fig. 2a). Likewise, T-bet gene expression in patients with high-grade bladder cancer was significantly higher than that in patients with low- and medium-grade disease (p = 0.005) (Fig. 2b). There were no statistical differences between patients with or without recurrence (Fig. 2c), or with or without progression (Fig. 2d), suggesting that T-bet was not a marker of progression or recurrence of patients with bladder cancer. However, its reduction may be of good prognostic value. It is known that T-bet is responsible for the expression of IFNγ, a cytokine secreted by Th1 [23] that exhibits anti-proliferative and pro-apoptotic mechanisms [24]. To determine whether T-bet positive cells are Th1, we evaluated the expression of interferon gamma (IFNγ) in tumors, by RT-qPCR. We found that there was no IFNγ expression in tumors, whatever their grade or stage (data not shown).

### GATA-3 expression in bladder cancer tissue

**Fig. 3:**
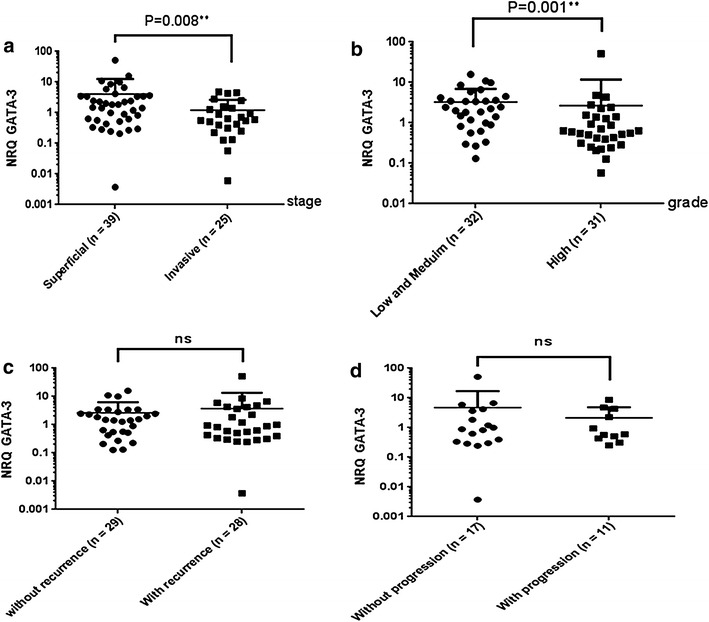
GATA-3 expression levels according to clinic-pathological features: **a** Statistical analyses of GATA-3 expression and pathological stage **p = *0.008*. **b** Statistical analyses of GATA-3 expression and pathological grade **p = *0.001*. **c** No correlation between GATA-3 expression and recurrence. **d** No correlation between GATA-3 expression and tumor progression

We next investigated by real-time PCR expression of the Th2-associated gene GATA-3 in bladder tumors. The expression level of GATA-3 in non-invasive carcinoma (Tis, Ta, and T1) was significantly higher (p = 0.008) than in invasive bladder tumors (T2–T4) (Fig. 3a). Likewise, GATA-3 gene expression in patients with low- and medium-grade cancers was significantly higher than that in patients with high-grade bladder carcinomas (p = 0.001) (Fig. 3b). On the other hand, GATA-3 levels were not associated with recurrence or progression of non-muscle-invasive tumors (Fig. 3c, d). Limited to phenotyping of Th2 by the expression of GATA-3, these results suggest that reduced Th2 phenotype correlates with disease aggressiveness and a poor prognostic value.

### Bcl-6 expression in bladder cancer tissue

**Fig. 4:**
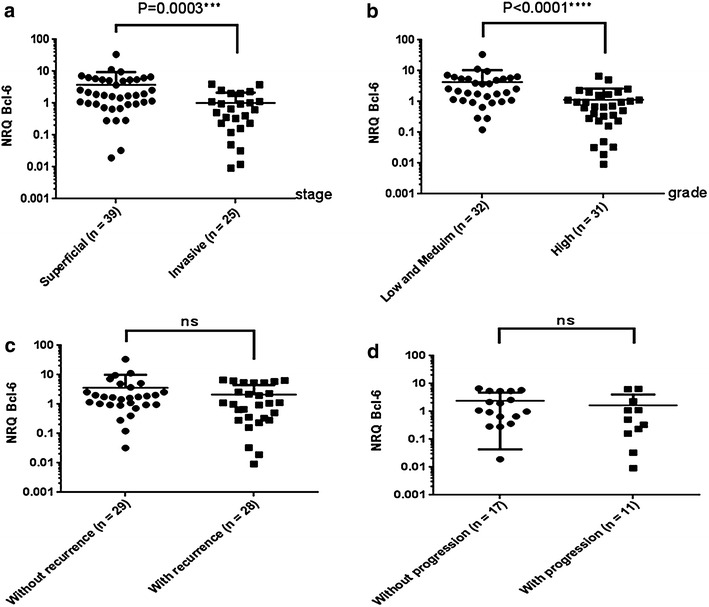
Bcl-6 expression levels according to clinic-pathological features: **a** Statistical analyses of Bcl-6 expression between superficial and invasive histological stages of bladder cancer ***p = *0.0003.* **b** Statistical analyses of Bcl-6 expression between low and high histological grades of bladder cancer ****p < *0.0001.* **c** No correlation between Bcl-6 expression and recurrence. **d** No correlation between Bcl-6 expression and tumor progression

We next assessed whether Bcl-6 gene expression in bladder cancer was correlated to the clinic-pathological features of patients. Real-time PCR analysis showed that Bcl-6 gene expression was higher in non-invasive bladder cancer tissue (Tis, Ta, and T1) than in invasive bladder tumors (T2–T4) (p = 0.0003) (Fig. 4a). Patients with low- and medium-grade carcinomas expressed the Bcl-6 gene to high levels compared to patients with high-grade disease (p < 0.0001) (Fig. 4b). Gene expression of the Bcl-6 gene was associated neither with disease progression nor with recurrence of bladder cancer tumors (Fig. 4c, d). Limited to the phenotyping of Tfh by the expression of Bcl-6, these results suggest that similarly to Th2, reduced tumor infiltration with Tfh cells correlates with disease aggressiveness and poor prognostic value.

### Survival analysis

**Fig. 5:**
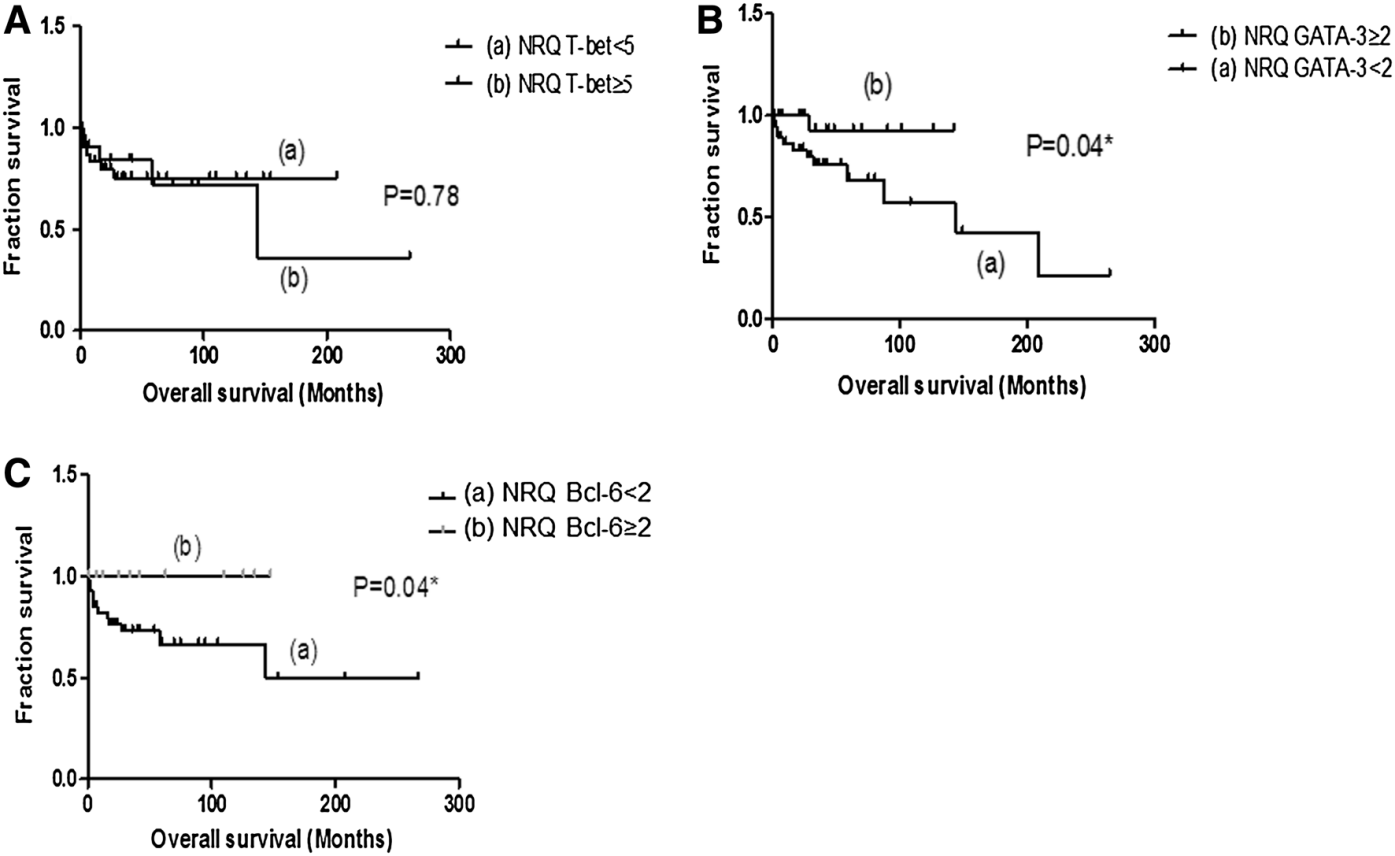
Kaplan–Meir plots for overall survival (*OS*) for T-bet, GATA-3, and Bcl-6 gene expression in patients with primary bladder cancer. Stratified survival curves for 65 patients by relative gene expression evaluated by qPCR. **A** Survival of patients with low and high T-bet expression levels. **B** Survival of patients with low and high GATA-3 expression levels *p = *0.04*. **C** Survival of patients with low and high Bcl-6 expression levels *p = *0.04*

Next, we analyzed T-bet, GATA-3, and Bcl-6 levels in relation to OS of patients with bladder cancer. No correlation was found between T-bet expression and patient survival (p = 0.78) (Fig. 5A). However, high expression of GATA-3 (Fig. 5B) and Bcl-6 (Fig. 5C) were associated with a better OS rate (p = 0.04 and p = 0.04, respectively).

### The prognostic value of T-bet, GATA-3 and Bcl-6 in the response to BCG treatment and in tobacco addicts

**Fig. 6:**
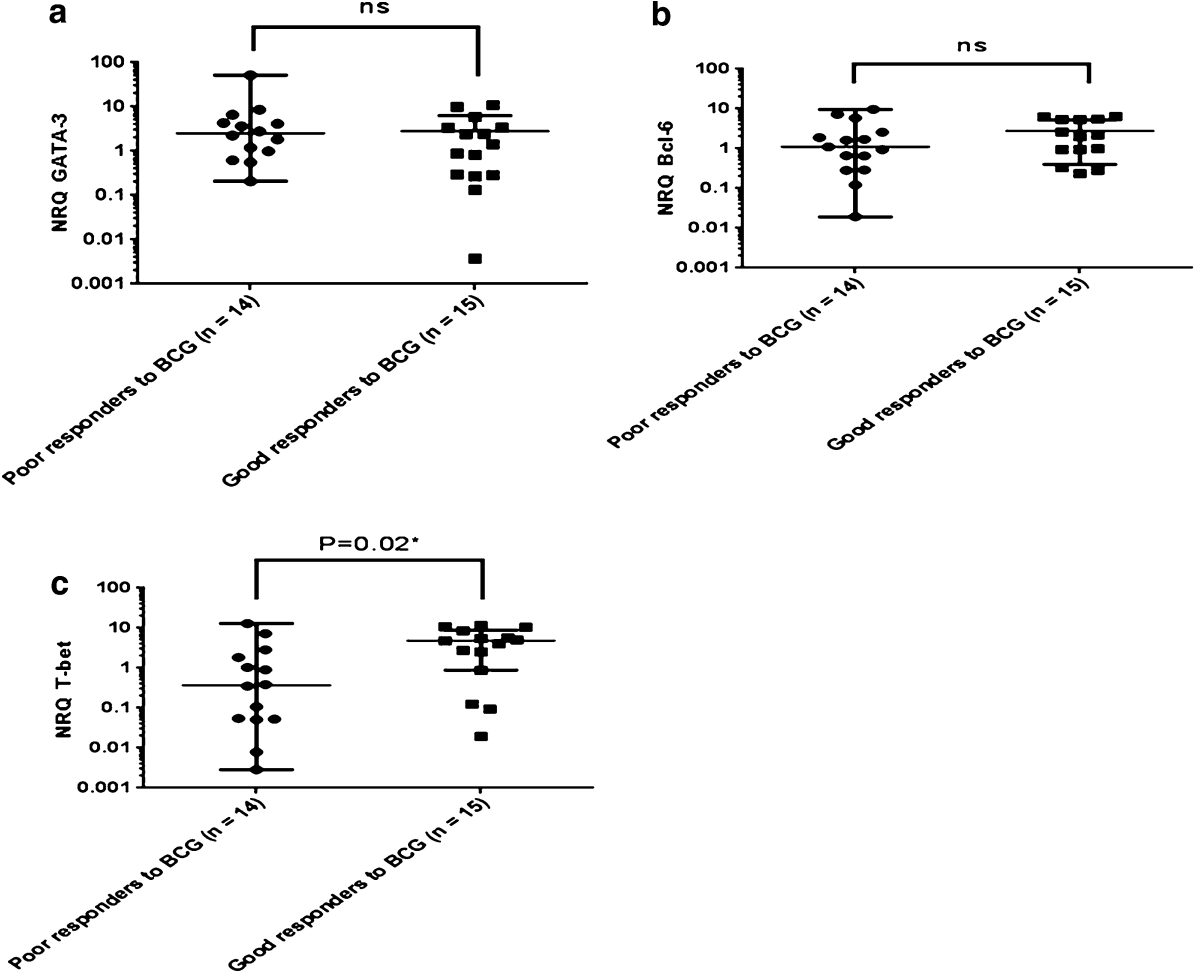
Correlation of T-bet, GATA-3 and Bcl-6 gene expression with response to BCG therapy. Expression of these transcription factors was evaluated by RT-qPCR. **a** Statistical analyses of GATA-3 expression for poor and good responders to BCG *p = *0.02.* **b** Statistical analyses of Bcl-6 expression for poor and good responders to BCG. **c** Statistical analyses of T-bet expression for poor and good responders to BCG

We further compared the expression of the three transcription factors, T-bet, GATA-3 and Bcl-6 to treatment with BCG, in good or poor BCG responders. Real-time PCR analysis showed that there is no significant difference between the patient response to BCG and the expression of GATA-3 (Fig. 6a) and Bcl-6 (Fig. 6b), whereas the expression of T-bet is higher among patients who respond well to BCG as compared to non-responders (p = 0.02) (Fig. 6c). These results suggest that T-bet might be a predictor of the response to BCG therapy in patients with bladder cancer.

**Fig. 7:**
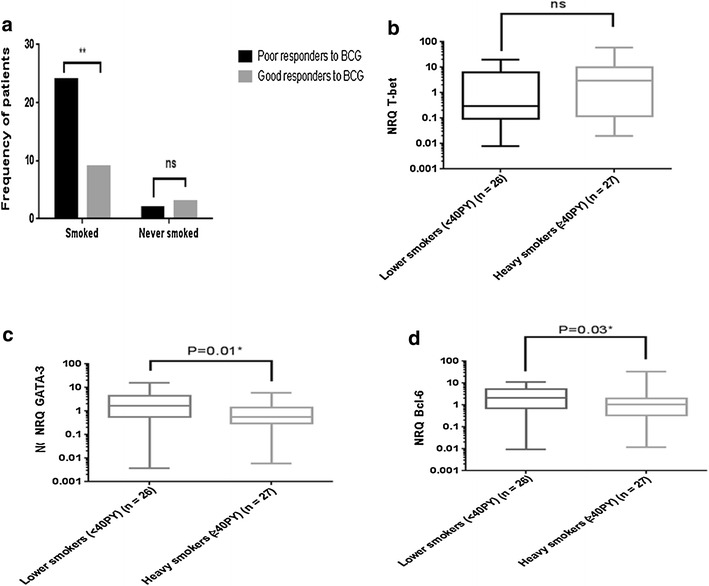
Correlation of T-bet, GATA-3 and Bcl-6 expression levels and intensity of tobacco consumption of patients with bladder cancer. **a** Frequency of smokers’ patients according the BCG response. **b** Statistical analyses of T-bet expression levels between heavy and light smoker patients. **c** Statistical analyses of GATA-3 expression levels between heavy and light smokers *p = *0.01*. **d** Statistical analyses of Bcl-6 expression levels between heavy and light smokers *p = *0.03. PY* packets per year

We also correlated the expression of these three genes with tobacco addiction of patients with bladder cancer. First, we showed that heavy smokers are poor responders to BCG (Fig. 7a). T-bet expression was not significantly different between heavy smokers and those who smoked less since their disease onset (Fig. 7b). In contrast, the expression of both GATA-3 (Fig. 7c) and Bcl-6 (Fig. 7d) was significantly lower in heavy smokers than in light ones (p = 0.01 and p = 0.03) respectively. These results suggest that smoking during treatment can be detrimental for disease outcome.

## Discussion

The present study showed differences in levels of immune-related transcription factors between groups of patients suffering from bladder cancer, according to the histopathological features of the disease. Our data showed that high levels of T-bet were associated with invasive carcinomas and patients with high-grade cancer. Although T-bet was originally cloned as a key transcription factor, involved in the commitment of T helper (Th) cells to the Th1 lineage, it plays multiple roles in many subsets of immune cells, including innate lymphoid cells or type 1 (ILC1), B cell, Th17 and dendritic cells [25]. It appears that these Th1 cells are not active in tumors, which explains their association with high-grade and stage cancer. In our study, we failed to detect IFNγ in tumors (data not shown). From this point of view, our results suggest that the increased expression of T-bet in the tumors in the absence of IFNγ may be considered as a marker of high-grade and advanced-stage disease. Interestingly, we found that T-bet expression correlated with a good response to BCG therapy. This result could be explained by the BCG reactivation of unreactive Th1-type cells present in tumors, which is expected to induce local inflammation. Our results agree with those that suggest that the BCG therapy response is correlated with the magnitude of T-cell infiltration in patients’ populations [26]. Using a mouse tumor model, several groups have reported that BCG-mediated antitumor activity requires a functional immune system, including CD4^+^ and CD8^+^ T lymphocytes [27]. Despite the characterization of biomarkers predicting a response to BCG immunotherapy, few are promising as predictive markers of a response to BCG treatment [28].

Our results showed that the expression of GATA-3, a well**-**defined regulator of T helper2 (Th2) cell differentiation, was significantly higher in superficial non-invasive carcinoma and in patients with low- or medium-grade disease. Our results did not allow the expression of GATA-3 to be associated with Th2, since we have not analyzed the expression of Th2-associated cytokines such as IL- 4, IL-5 and IL-13. It is also known that GATA-3 regulates the specification and differentiation of many cell and tissue types, including: innate lymphoid cells (ILC), adipose tissue, endothelial cells, urothelium (a constituent of the bladder tumor environment) [29, 30]. Although we were unable to associate the expression of GATA-3 to Th2 or another type of cell, it remains possible that this transcription factor serves as a good prognosis biomarker in bladder cancer, since its expression is associated with patient survival. We observed an inverse correlation between GATA-3 and T-bet expression in tumors. More precisely, in each sample, when T-bet expression was high, GATA-3 was low and vice versa. We can thus propose the hypothesis that the presence of Th1-type cells limits that of Th2-type. Evidence has accumulated that cancer patients with non-small cell lung cancer [31], oral cancer [32], cervical cancer [33], and bladder cancer [34] have an imbalance in the Th1/Th2 axis. However, in these later reports, patients develop Th2 dominant status with deficiency in Th1. In the present study, we not observed prevalence of status relative to each other, but rather the presence of one affects the expression of the other. In our results, no correlation was observed with GATA-3 expression and BCG response. Nunez-Netras et al. [35] reported controversial results, showing correlation between high GATA-3 expression and good BCG response. This discrepancy could be related to the *s*mall number of patients and more particularly that all patients exhibited Tis stage, unlike our patients that had various stages and grades of the disease.

We also examined the expression of Bcl-6, which has been defined as an essential element for the development of Tfh cells. This transcription factor has been described to be highly expressed in various human cancers. We found that the level of Bcl-6 was significantly higher in non-invasive carcinoma and in low- and medium-grade tumors than in invasive and high grade. Although overexpression of Bcl-6 was frequent in diffuse large B cell lymphoma [36], its involvement in other cancer types was controversial. Our study is consistent with a report indicating that Bcl-6 expression was significantly increased in transitional cell carcinoma of the urinary bladder, kidney and urethra [37]. In addition, Bcl-6 protein expression, determined by immunohistochemical staining, correlated positively with grades 1 and 2 but not grade 3 of transitional cell carcinomas [37]. If we consider that the cells that express Bcl-6 are largely Tfh, our results are consistent with those indicating that impaired function of Tfh cells is associated with hepatocellular carcinoma progression [18]. Recent data also revealed that a detectable Tfh cell presence is associated with long-term positive clinical outcome in patients with either breast or colon cancer [17, 38]. Conversely, further studies revealed that Bcl-6 expression was associated with disease progression and poor survival of breast cancer patients [16]. To assign Bcl-6 to Tfh required the expression of markers associated with Tfh, such as: CXCR5, CXCL13 and IL-21 to be assessed. Considering the expression of Bcl-6 only in tumors, we can suggest that the expression of this transcription factor is associated with a good prognosis in bladder cancer.

We also showed that the expression of both GATA-3 and Bcl-6 but not T-bet was significantly lower in heavy smokers than in light ones, and heavy smokers are poor responders to BCG. Cigarette smoking is the best established risk factor for the development of bladder cancer and disease progression [39]. Many of the adverse effects of smoking might result from the ability of cigarette smoke to suppress the immune system [40]. To our knowledge, our study is the first to correlate tumor-infiltrating T cells to smoking in bladder cancer. It is worthy to note that among high smokers, the good BCG responders exhibited high T-bet expression, which is consistent with the good response to BCG of patients with high T-bet expression.

## Conclusions

In summary, our results suggest that T-bet can be used as a predictive marker for good responses to BCG therapy. This conclusion could explain the fact that T-bet expression is not correlated with recurrence, progression nor survival, even though it is associated to high-grade and -stage in the diagnosis of bladder cancer, whereas an increase of GATA-3 and Bcl-6 expression are associated with a good survival rate. More detailed characterization of cell populations expressing these transcription factors might provide opportunities to eliminate protumoral cells in bladder cancer or harness antitumoral cells or compounds.

## Abbreviations

BCG: bacillus calmette-guerin
Bcl-6: B-cell lymphoma 6
GATA-3: trans-acting T cell-specific transcription factor 3
Tfh: T-cell follicular “helper”
T-bet: T-box transcription factor TBX21
TNM: tumor node metastasis
WHO: World Health Organization
